# Bariatric Surgery Modulates Urinary Levels of MicroRNAs Involved in the Regulation of Renal Function

**DOI:** 10.3389/fendo.2019.00319

**Published:** 2019-05-21

**Authors:** Abdullah Alkandari, Hutan Ashrafian, Thozhukat Sathyapalan, Ara Darzi, Elaine Holmes, Thanos Athanasiou, Stephen L. Atkin, Nigel J. Gooderham

**Affiliations:** ^1^Department of Surgery and Cancer, Imperial College London, London, United Kingdom; ^2^Dasman Diabetes Institute, Kuwait City, Kuwait; ^3^Institute of Global Health Innovation, Imperial College London, London, United Kingdom; ^4^Department of Academic Endocrinology, Diabetes, and Metabolism, Hull York Medical School, Kingston upon Hull, United Kingdom; ^5^Weill Cornell Medical College Qatar, Qatar Foundation, Doha, Qatar

**Keywords:** microRNA, nephropathy, bariatric/metabolic surgery, urinary, longitudinal, diabetes

## Abstract

**Background:** Obesity and diabetes cause chronic kidney disease with a common pathophysiology that is characterized by the accumulation of collagen in the extracellular matrix. Recent evidence has implicated the epithelial-to-mesenchymal transition (EMT) as a key step in this pathology with regulation by microRNAs. Weight loss leads to improvements in renal function; therefore, this study hypothesized that bariatric-surgery aided weight loss would lead to changes in urinary microRNAs involved in the regulation of renal function.

**Materials and methods:** Twenty-four bariatric patients undergoing Roux-en-Y gastric bypass and sleeve gastrectomy donated urine pre-operatively and at 2–6 months and 1–2 years post-operatively. Urine samples were also obtained from 10 healthy weight and 7 morbidly obese non-surgical controls. Expression levels of kidney microRNAs were assessed in urine and the function of microRNAs was assessed through the *in vitro* transfection of HK-2 cells, a kidney proximal tubule cell line.

**Results:** Levels of miR 192, miR 200a, and miR 200b were upregulated in urine following bariatric surgery. This increase was consistent across surgical type and diabetes status and was maintained and enhanced with time. Bariatric surgery alters urinary miR 192 expression from levels seen in morbidly obese patients to levels seen in healthy weight control patients. In mechanistic studies, the transfection of miR 192 in HK-2 cells increased miR 200a expression and decreased ZEB2, a key transcriptional promoter of kidney fibrosis.

**Conclusions:** Bariatric surgery increased miR 192 and miR 200 urinary levels, key anti-fibrotic microRNAs that could contribute to a renal-protective mechanism and may be of value as urinary biomarkers following surgery. These findings suggest that urinary microRNAs may represent potential novel biomarkers for obesity-associated renal function.

## Introduction

Whilst obesity contributes to diabetes and hypertension, both leading causes of progressive chronic kidney disease (CKD), data suggests that obesity is an independent risk factor for CKD (1). Diabetes and obesity related CKD appear to share the same initial pathophysiology (1). Increasing BMI leads to renal hyperfiltration and proteinuria that eventually culminates in hypofiltration and CKD. Diabetic nephropathy (DN) occurs in approximately one third of cases of diabetes (both type 1 and type 2) and is the most common cause of CKD and end stage renal failure (2). DN is mediated by transforming growth factor-β1 (TGF-β1) and TGF-β1 promoted fibrosis is induced by increasing the number of local fibroblasts, which are the effecter cells in the production and accumulation of the extracellular matrix. Epithelial-to-mesenchymal transition (EMT) is increasingly recognized as a key component in this process by promoting the transition of resident renal epithelial cells to a fibroblast phenotype (3). Recently, multiple studies have implicated microRNAs in this process. MicroRNAs are small, endogenous RNAs that regulate gene expression by silencing the translation of mRNA. The microRNAs miR 192 and the miR 200 family contribute to kidney fibrosis by suppressing EMT, targeting zinc E-box binding homeobox 1 and 2 (ZEB1 and ZEB2) that encode for transcriptional repressors for E-cadherin, essential for maintaining the epithelial phenotype (4–6). This leads to an increase in collagen deposition in the extracellular matrix and the thickening of the glomerular basement, a common feature of DN.

Weight loss in the clinically obese patient, including that achieved surgically, leads to an improvement in renal function (7–9). Bariatric or metabolic surgery encompasses several gastrointestinal surgical procedures that offer metabolic modulation to diminish metabolic dysfunction and induce substantial weight loss through the BRAVE physiological steps–bile flow alteration, reduction of gastric size, anatomical gut rearrangement, and altered flow of nutrients, vagal manipulation, and enteric gut hormone modulation (10). The two most common bariatric procedures worldwide are the Roux-en-Y gastric bypass (RYGB) and the sleeve gastrectomy (11). In a RYGB, a small gastric pouch (15–30mL) is created and separated from the larger stomach remnant. The jejunum is then incised from the duodenum and anastomized to the small stomach pouch forming a distinctive “Y” shape that gives this procedure its name (12). In a sleeve gastrectomy, a longitudinal gastric sleeve is created running from the esophagus to the small intestine that is separated from the larger excised stomach (13). In comparison to non-surgical strategies, bariatric surgery is more effective at achieving sustained weight loss, improved quality of life and increased life expectancy (14, 15). Several studies have shown that bariatric surgery also leads to remission or resolution of type 2 diabetes and improved glycaemic control (16–19). These improvements are observed early post-operatively, before substantial weight loss and to a greater extent than in patients who achieve similar weight loss non-surgically, and are therefore, thought to offer weight-independent metabolic effects (20, 21) in addition to weight-dependent metabolic actions. Hypothesizes for this phenomenon include the foregut, hindgut, and midgut theories (22), but the precise mechanisms behind improvement of diabetes following bariatric surgery are still not fully understood. Bariatric surgery also leads to improvements in cancer risk, sleep apnoea, and other obesity-associated disorders (23, 24). Thus, bariatric surgery may target and improve renal function through direct mechanisms of weight loss as well as through diabetes dependent mechanisms.

MicroRNAs have attracted a great amount of recent attention as potential novel biomarkers of disease due to their stability in biofluids and their facile detection through PCR and profiling platforms (25, 26). Circulating microRNAs are reported biomarkers for cancer, obesity, and diabetes (25, 27, 28), and distinct bariatric microRNA profiles have been characterized in circulation (28, 29). Urinary microRNAs are reported biomarkers for immunoglobulin A nephropathy and bladder cancer (30, 31). Currently the non-invasive diagnosis of CKD relies on urinary microalbuminuria and estimated glomerular filtration rate (eGFR). However, the sensitivity and specificity of these markers are poor, particularly in obese patients, and only manifest late in the disease process.

We hypothesized that bariatric surgery modulates the urinary levels of microRNAs involved in the regulation of renal function. Therefore, we aimed to study urinary expression of renal microRNAs in bariatric patients before and after surgery in comparison to non-surgical control subjects.

## Materials and Methods

### Study Design

Urine samples were collected for 10 healthy weight controls, seven morbidly obese controls and 24 bariatric surgical patients in Hull and East Yorkshire Hospitals and Imperial College Healthcare NHS Trusts under ethics approvals 09/H1304/46 and 08/H0711/123. Informed written consent was provided by all participants enrolled in this study and all appropriate guidelines were followed when conducting the methods. For the bariatric cohort, urine samples were obtained pre-operatively and at two periods post-operatively–between 2 and 6 months and between 1 and 2 years (Table 1). Nine out of the 24 bariatric patients had diabetes pre-operatively. Bariatric surgery was performed laparoscopically. Twenty bariatric patients had a RYGB and four had a sleeve gastrectomy. All bariatric patients fulfilled NICE qualifying criteria for bariatric surgery (32). All subjects had normal serum urea, creatinine, and eGFRs at baseline.

**Table 1:** Study summary.

	**Controls**		**Bariatric cohort**		
	**Healthy**	**Morbidly obese**	**RYGB + Sleeve**	**RYGB**	**Sleeve**
*n*	10	7	24	20	4
Age	39.1 (16.3)	54 (11.7)	45.7 (10.8)	45 (10.4)	49.1 (13.7)
Female (*n*)	8	3	16	14	2
Diabetes (*n*)	0	1	9	8	1

† *Values represent mean. Standard deviation in parenthesis*.

### Urinary RNA Extraction

RNA was extracted from 250 μl of urine using the *mir*Vana PARIS kit (Ambion, Paisley, UK) according to the manufacturer's instructions with the one modification; to normalize and for carrier purposes, 30 femtomoles of synthetic *C. elegans* miR-39 (Ambion) was spiked into the urine samples prior to the acid-phenol:chloroform phase. Cel. miR-39 was chosen as carrier as it lacks sequence homology with human miRNAs. RNA was eluted in 100 μl nuclease free water.

### Cell Culture

The human kidney proximal tubule cell (PTC) line HK-2 was obtained from ATCC (LGC Standards, Teddington, UK) and maintained in DMEM/Ham's F12, supplemented with 10% v/v fetal bovine serum (FBS), 1% Penicillin/Streptomycin antibiotics (100 U/ml, 100 μg/ml, respectively), and 1% Insulin-Transferrin-Sodium Selenite (Sigma-Aldrich, Gillingham, UK) as previously described (6).

### Transfections

Endogenous microRNA levels were manipulated in HK-2 cells through the transfection of microRNA miR 192 mimic, a gain-of-function experiment designed to determine the biological activity of miR 192. mIRIDIAN microRNA mimics and mimic controls were purchased from Dharmacon (Lafayette, CO, USA). The siPORT NeoFX transfection agent was used according to the manufacturer's instructions. HK-2 cells at approximately 80% confluence were trypsinized and seeded at 50,000 cells per well in complete media in 24 well cell culture plates and transfected with miR-192 mimic at 10, 20, and 50 nM concentrations for 48 h. Following SiPORT transfection RNA was extracted from HK-2 cells using the *mir*VANA PARIS kit according to manufacturer's instructions and eluted in 75 μl nuclease free water.

### Quantitative PCR

MicroRNA was reverse transcribed into cDNA using the TaqMan MicroRNA Reverse Transcription kit (Applied Biosystems, Paisley, UK) and messenger RNA (mRNA) was reverse transcribed using the High Capacity Reverse Transcription kit (Applied Biosystems) according to manufacturer's instructions. Relative microRNAs and mRNA levels were determined by real time, quantitative PCR using TaqMan Fast Universal Master Mix (Applied Biosystems). Quantitative PCR was performed in triplicate on the Applied Biosystems 7,500 Fast System. All PCR and RT probes were obtained from Applied Biosystems. Urinary microRNA expression was first normalized to creatinine to correct for diuretic effect (Creatinine Assay, R & D Systems, Abingdon, UK) and then normalized to cel. miR-39. Cellular microRNA expression following HK-2 transfections was normalized to U6 small nuclear RNA and mRNA expression was normalized to GAPDH, a frequently used housekeeping gene. Relative expression was calculated using the 2^−ΔCt^ method (33).

### Statistical Analysis

Urinary microRNA data is expressed as fold change relative to pre-operative levels or morbidly obese control levels. Data is represented as mean ± standard error of mean (SEM). Statistical significance was determined using ANOVA (with a post-test for linear trend) and paired/unpaired Student's *t*-test where appropriate. Significance was considered as *p* < 0.05. Statistical analysis was performed using GraphPad Prism 6.0 (Graphpad, La Jolla, CA, USA).

## Results

### Bariatric Clinical Outcomes

**Figure 1:**
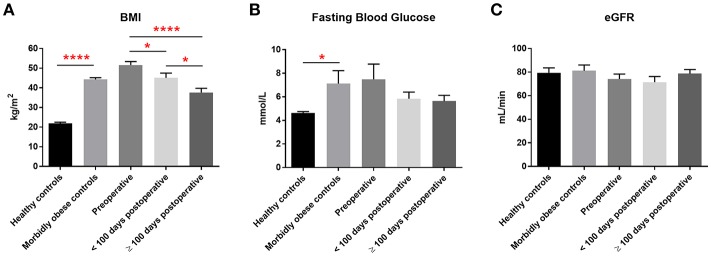
Clinical measurements. Pre-operative, post-operative, and control levels of **(A)** BMI, **(B)** fasting blood glucose levels and **(C)** eGFR. Data represent mean ± SEM. ^*^p < 0.05, ^****^p < 0.0001 by Student's *t*-test.

Bariatric surgery induced substantial clinical improvements in all bariatric patients (Figure 1). Reduction in BMI was significant within 100 days of surgery and mean BMI decreased by almost 30% at 100 days or more after surgery. Mean levels of fasting blood glucose also decreased following bariatric surgery, decreasing from 7.5 mmol/L pre-operatively to 5.6 mmol/L 100 days or more following surgery, although this decrease was not statistically significant. eGFR following bariatric surgery was unchanged (mean 74–79 mL/min).

### Bariatric Surgery Increases Urinary miR 192 Levels

**Figure 2:**
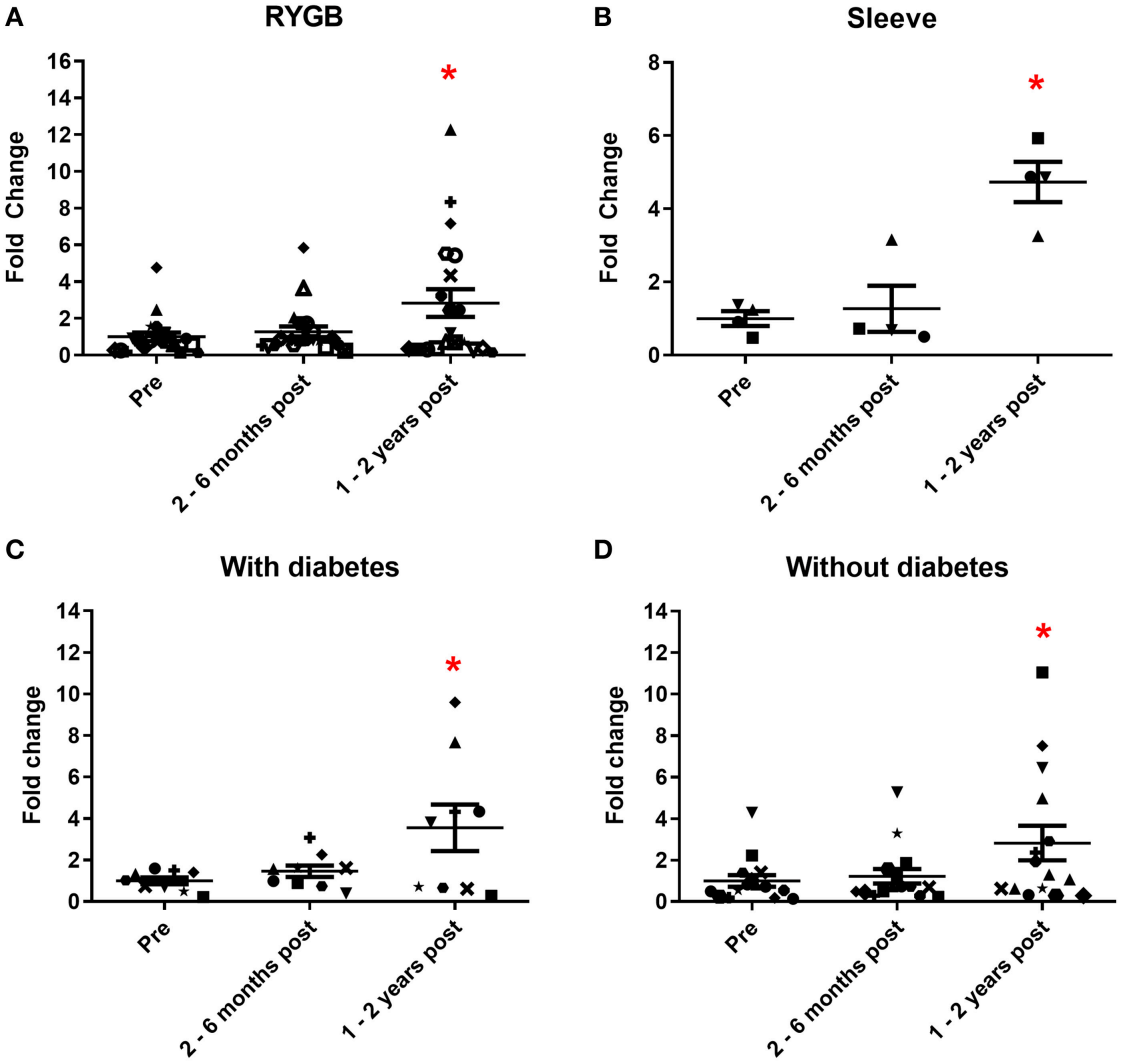
Urinary miR 192 expression following bariatric surgery. **(A)** RYGB patients. **(B)** Sleeve gastrectomy patients. **(C)** Patients with diabetes. **(D)** Patients without diabetes. Data represents mean fold change ± SEM relative to mean preoperative expression. *n* = 20 for RYGB, *n* = 4 for sleeve, *n* = 9 for diabetes, and *n* = 15 for individuals without diabetes. Each patient is represented by a unique symbol. ^*^p < 0.05 vs. preoperative expression by paired *t*-test.

We initially assessed the urinary expression of miR 192, a consistently abundant renal microRNA (34). In patients who had laparoscopic RYGB, levels of miR 192 significantly increased 1.3 and 2.9-fold (relative to pre-operative levels) at 2–6 months and 1–2 years, respectively, following surgery (Figure 2A). A similar increase was observed in sleeve gastrectomy patients. MiR 192 levels increased 1.3-fold at 2–6 months following sleeve procedures and 4.7-fold (significant relative to pre-operative levels) at 1–2 years post-operatively (Figure 2B). Increase in urinary miR 192 levels was consistent when patients were stratified by pre-operative diabetes status. In patients with diabetes urinary levels of miR 192 increased 1.5-fold at 2–6 months and significantly 3.6-fold at 1–2 years following bariatric surgery (Figure 2C). Patients without diabetes demonstrated a significant 2.8-fold increase in miR 192 levels at 1–2 years following surgery (Figure 2D).

**Figure 3:**
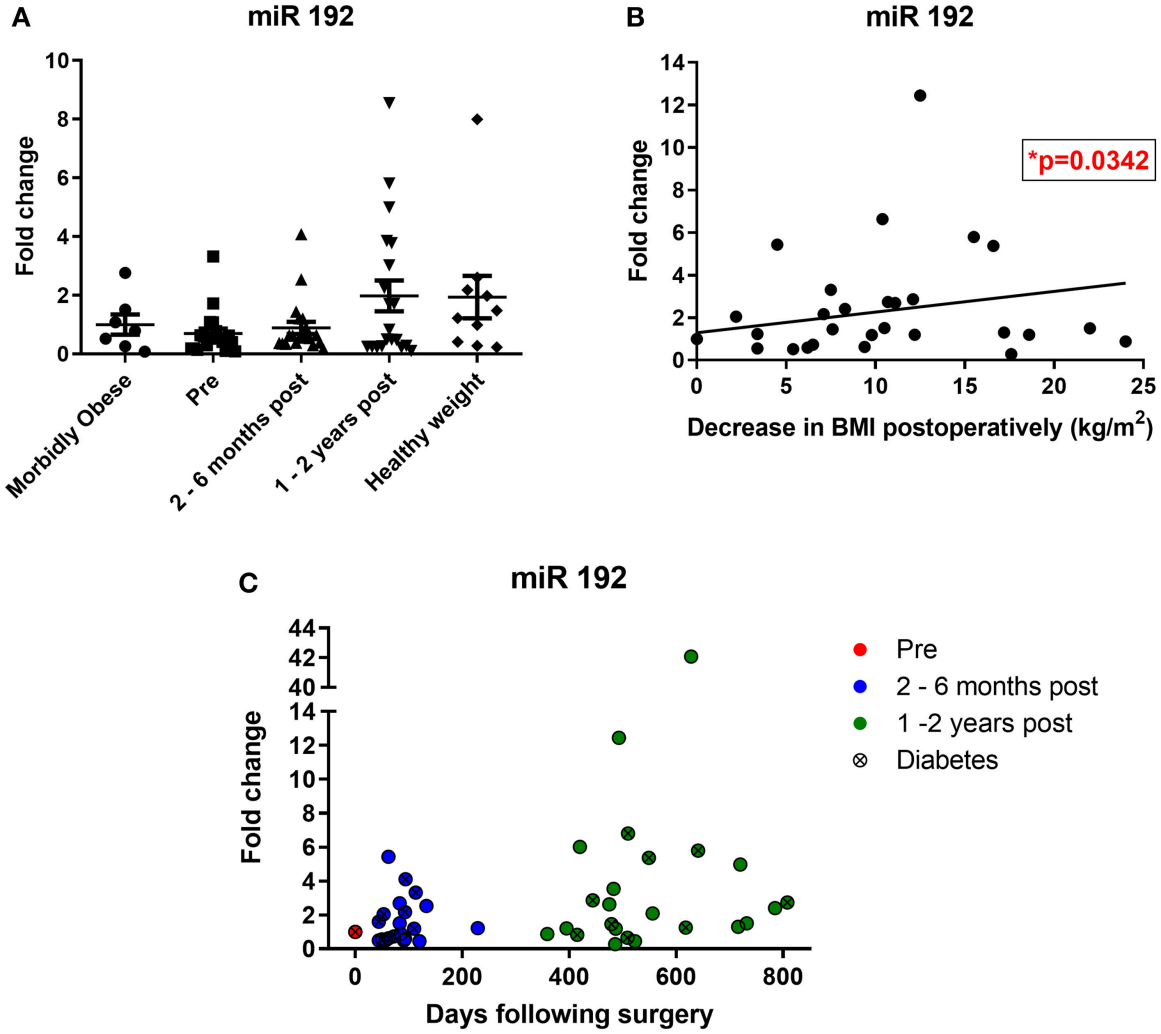
Bariatric urinary miR 192 expression relative to a morbidly obese cohort. **(A)** Bariatric and healthy weight miR 192 expression relative to a morbidly obese control group. Data represents mean ± SEM relative to mean preoperative expression. Morbidly obese is a BMI ≥ 40 kg/m^2^, healthy weight is a BMI < 25 kg/m^2^. *n* = 7 for morbidly obese, *n* = 10 healthy weight, *n* = 24 for preoperatively, 2–6 months and 1–2 years post-operatively. **(B)** Pearson correlation of miR 192-fold change against change in post-operative BMI. *n* = 46. **(C)** Timecourse of miR 192 expression following surgery. Data represented as fold change relative to individual preoperative expression for 24 bariatric patients.

When expressed as fold change relative to the morbidly obese control group, bariatric pre-operative miR 192 levels were lower (0.6-fold), potentially reflecting the higher BMI in patients undergoing bariatric surgery (Figure 3A). Bariatric miR 192 expression was slightly higher 2–6 months following surgery (0.8-fold relative to morbidly obese controls). However, 1–2 years following bariatric surgery, mean miR 192 level was nearly 2-fold higher relative to the morbidly obese cohort and was comparable to miR 192 levels in those with a healthy weight (BMI under 25, Figure 3A). Although comparative two-way fold changes were not significant, the linear trend with time was (*p* = 0.0117, one-way ANOVA). An inverse correlation between miR 192-fold change and change in BMI post-operatively was also significant (*p* = 0.0342, Pearson's correlation, Figure 3B). The data show that bariatric surgery shifts urinary miR 192 expressions from levels observed in a morbidly obese population to levels observed in a healthy weight population.

Of the 24 individuals who made up the bariatric cohort, 20 had increased urinary miR 192 expression post-operatively relative to their pre-operative levels, including 16 out of the 20 RYGB patients, eight out of nine patients with diabetes and all four sleeve gastrectomy patients. A timecourse of post-operative miR 192 expressions in RYGB and sleeve patients relative to their individual pre-operative miR 192 levels can be found in Figure 3C. A significant correlation was found between individual, personalized fold changed in miR 192 levels and days following surgery (*p* = 0.0051, Pearson's correlation). This illustrated a trend of a gradual, general increase in miR 192 levels in urine which is enhanced and sustained with time and consistent with migration toward the healthy weight control phenotype.

### Bariatric Surgery Increases Urinary Levels of miR 200a and miR 200b

**Figure 4:**
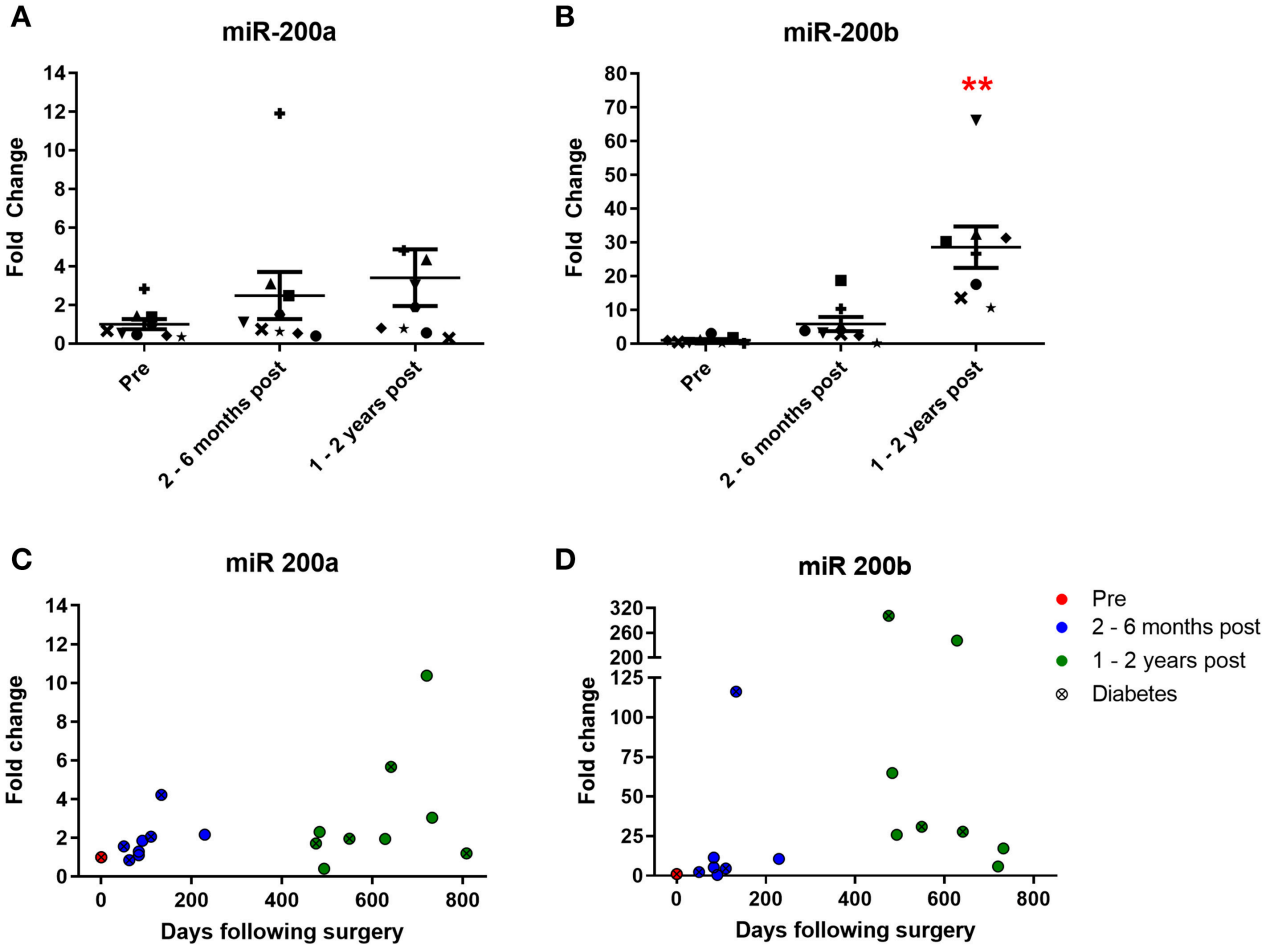
Urinary expression of miR 200 following bariatric surgery. **(A)** miR-200a, *n* = 9 **(B)** miR-200b, *n* = 8. Data represents mean fold change ± SEM relative to mean preoperative expression. ^**^p < 0.01 vs. preoperative expression by paired *t*-test. Each patient is represented by a unique symbol. Timecourse of **(C)** miR 200a and **(D)** miR 200b expression following surgery. Data represented as fold change relative to individual preoperative expression.

Urinary levels of miR 200a and miR 200b were also measured. Both are expressed in the kidney and change in their expression is associated with diabetic nephropathy (5, 6). MiR 200a urine levels increased 2.5-fold at 2–6 months and 3.4-fold at 1–2 years following surgery (Figure 4A), although neither increase achieved statistical significance. MiR 200b levels increased 5.8-fold at 2–6 months and strikingly 28.6-fold (significantly different from the pre-surgery levels) at 1–2 years post-operatively (Figure 4B). All but one patient demonstrated a post-operative increase of urinary miR 200a and all patients demonstrated an increase in urinary miR 200b relative to their individual pre-operative level. Significant correlations were observed between both miR 200a and miR 200b and days following bariatric surgery (Figures 4C,D, *p* = 0.0071 and 0.044, respectively, Pearson's correlation). There were also significant correlations between urinary levels of miR 192 and both miR 200a and miR 200b (Supplementary Figure 1, *p* = 0.0364 and *p* < 0.0001, respectively, Pearson's correlation).

### Mechanistic Studies in the Human PTC Line, HK-2

**Figure 5:**
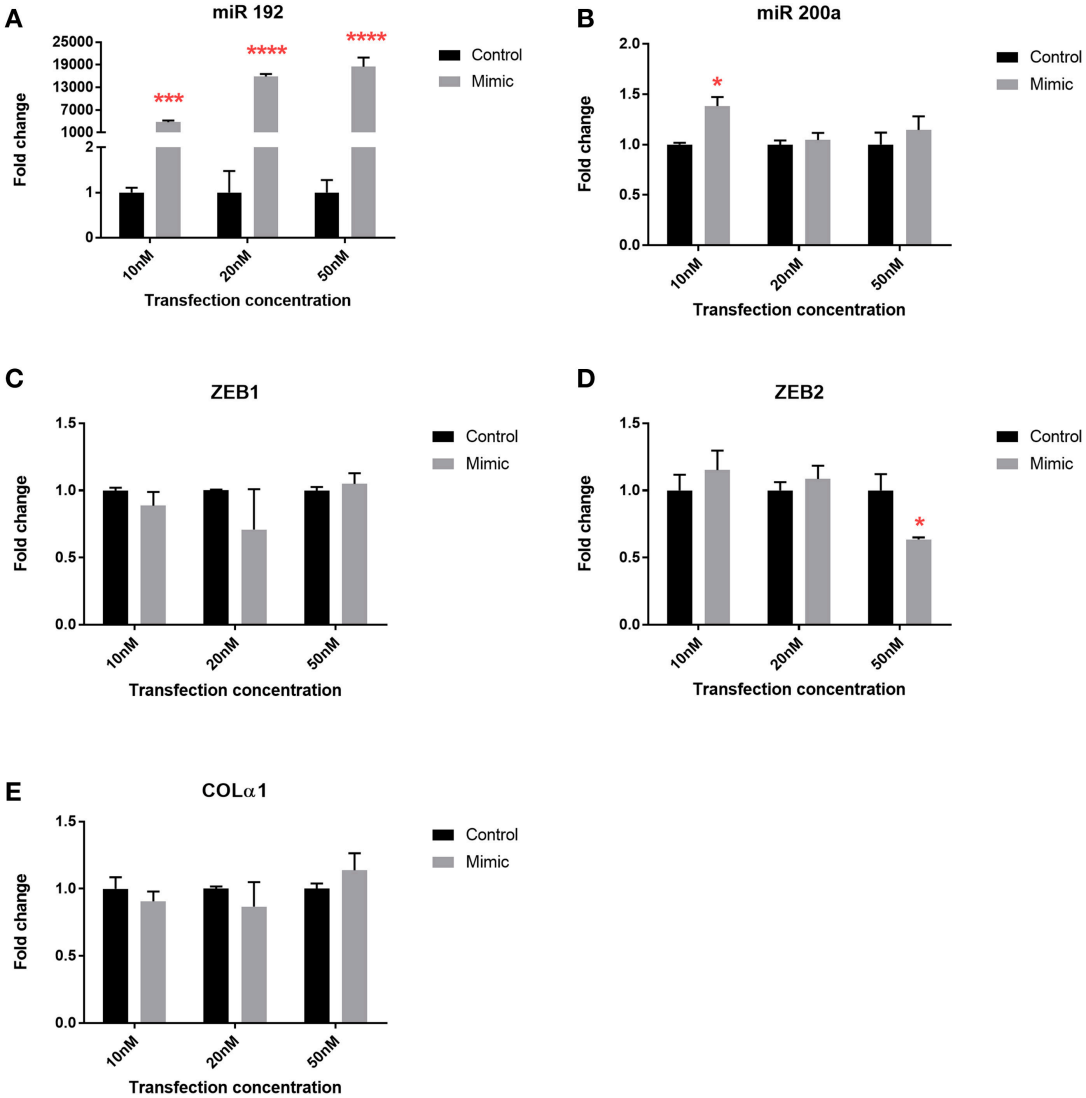
miR 192 mimic transfection of HK2 cells illustrates its anti-fibrotic role. Expression of **(A)** miR 192 **(B)** miR 200a **(C)** ZEB1 **(D)** ZEB2 and **(E)** Colα1 following 48 h of 10, 20, and 50 nM miR 192 mimic transfection. Data represents mean ± SEM relative to transfection with a scrambled control. ^*^p < 0.05, ^***^p < 0.001, ^****^p < 0.0001, *n* = 3.

To explore the mechanistic implications of altered microRNA expression following bariatric surgery, we used the human PTC HK-2 renal cell line to examine changes in phenotype. Transfection with all three concentrations of miR 192 mimic increased miR 192 expression 4,000, 16,000, and 18,000-fold, respectively, relative to cells transfected with a scrambled control (Figure 5A). Transfection of miR 192 increased levels of miR 200 at all concentrations, although the increase was only significant at 10 nM (Figure 5B). Expression levels of ZEB1 and Colα1 were unaltered for all three miR 192 mimic concentrations (Figures 5C,E). However, transfection with 50 nM miR 192 mimic concentration resulted in a significant 0.6-fold decrease in ZEB2 (Figure 5D).

## Discussion

There is a growing body of evidence that biofluid miRNA expression can be indicative of disease progression and prognosis (35–37). Urinary expression of microRNAs has also been proposed as useful in the diagnosis and prognosis of renal disease (38). We therefore considered whether bariatric surgery modulates urinary levels of miRNAs that regulate kidney function.

Three kidney-expressed microRNAs, miR 192, miR 200a, and miR 200b, were shown to be upregulated in urine following bariatric surgery, an increase that was consistent across surgical type and diabetes status and was sustained and enhanced with time. Bariatric surgery shifts urinary miR 192 expression from levels seen in morbidly obese patients to levels comparable with those seen in a healthy cohort. In mechanistic studies, the transfection of miR 192 in a human kidney PTC cell line increased miR 200a and decreased ZEB2, a key transcriptional promoter of kidney fibrosis. These three microRNAs assessed in urine could potentially represent non-invasive, anti-fibrotic biomarkers for surgery-induced improvement in renal function.

**Figure 6:**
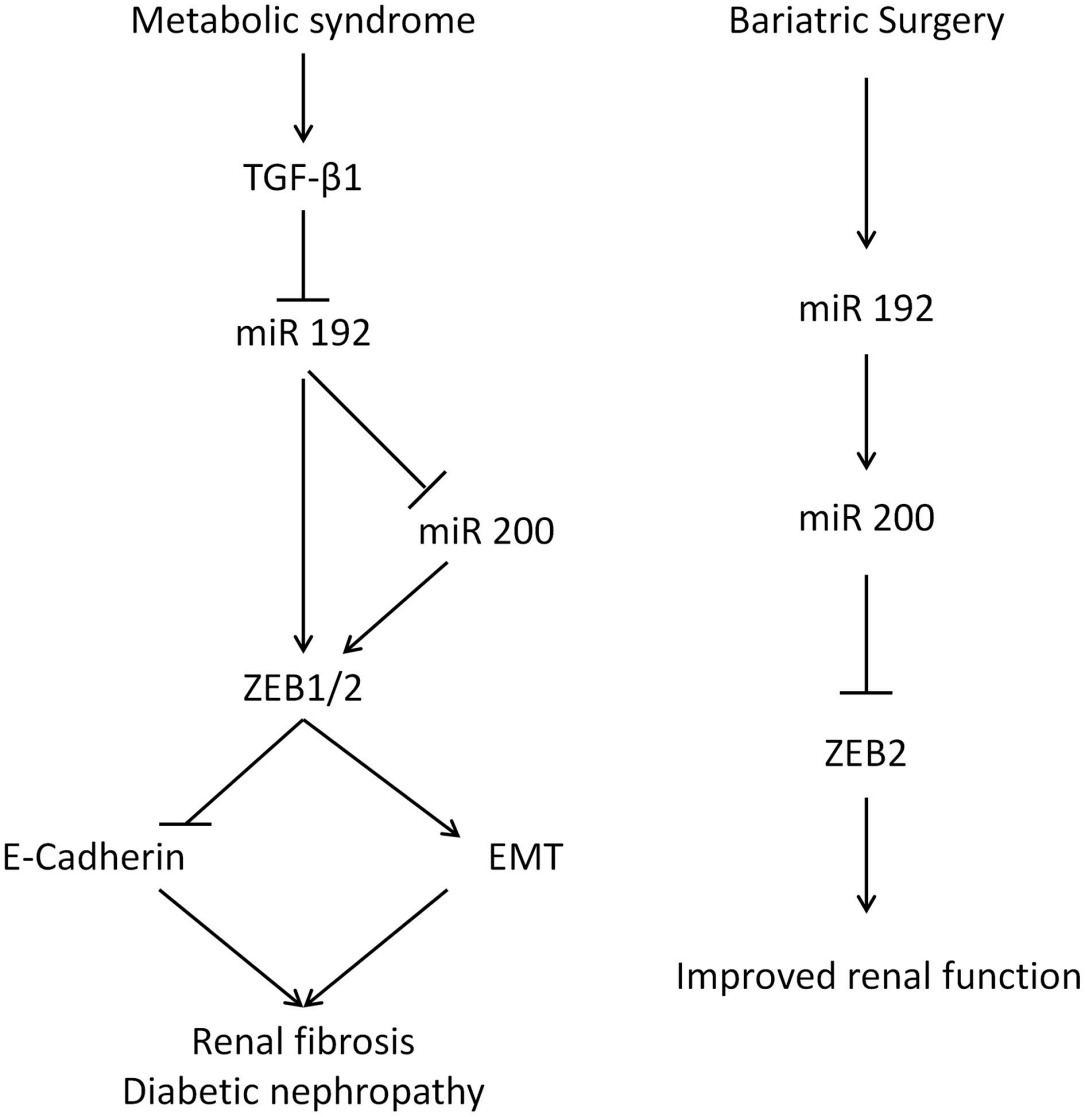
The renal function of miR 192 and miR 200. (**Left**) Mechanism for glucose-mediated renal fibrosis in the proximal tubular cells. Diabetes increases TGF-β which inhibits miR 192/200, lifting the suppression on ZEB and EMT. (**Right**) Suggested mechanism following bariatric surgery.

The kidney-specific deletion of Dicer, a ribonuclease involved in microRNA biogenesis, leads to proteinuria, glomerular disease, and tubular injury in mice (39–41). This illustrates the important functional roles of microRNAs in kidney pathophysiology. MiR 192 is one of 5 microRNAs whose expression is higher in the kidney relative to other organs (34), and its levels are reduced in human renal biopsies from patients with advanced diabetic nephropathy, levels that correlate with tubulointerstitial fibrosis, and decreased GFR, reflecting miR 192's key anti-fibrotic role (6). *In vitro* incubation of PTCs with TGF-β1 decreases miR 192 expression and the overexpression of miR 192 suppresses ZEB1 and ZEB2, decreasing TGF-β1 mediated E-cadherin suppression (6). In diabetic apoE mice, miR 192 expression was downregulated in the kidney and treatment of PTCs with TGF-β1 results in a reduction of miR 192 (42). Renal levels of the miR 200 family are decreased in mouse models of renal scarring and by TGF-β1 in rat PTCs (43). Injection of miR 200b precursor antagonizes ZEB1/ZEB2 induction, decreases collagen synthesis, and ameliorates fibrosis (44). p53 tumor suppressor increases expression of miR 192 and miR 200, suppressing EMT (45). It has also been reported that miR 200 expression is a strong biomarker for epithelial vs. mesenchymal phenotype in cancer (46). MiR 200 overexpression results in mesenchymal to epithelial transition in mouse carcinomas and overexpressing miR 200 *in vitro* increases E-cadherin and represses EMT (4, 5). Here, miR 192 transfection of human PTCs led to an increase in miR 200a, indicating that miR 192 could act upstream to miR 200 in a hierarchical regulation pathway, suggesting miR 192 may be a master microRNA regulator in the kidney (Figure 6). Previously miR 192 has been shown to upregulate miR 200b/c expression in mesangial cells (47).

In contrast to their roles in PTCs, miR 192, and miR 200 appear to have pro-fibrotic effect in mesangial cells. TGF-β1 increases miR 192 in mouse mesangial cells and repression of ZEB1/ZEB2 expression by miR 192 leads to collagen synthesis (48). The inhibition of miR 192 in streptozotin-induced diabetic mice reduces proteinuria (49). In mesangial cells, miR 200 family represses ZEB1/ZEB2, promoting fibrosis (47). The pleiotropic, cell-specific roles miR 192 and miR 200 play in renal fibrosis reflect the complex physiology of the kidney. Indeed, the renal cortex and the medulla have different microRNA expression profiles (50). It is worth noting that proximal tubular cells are further along the urinary tract than mesangial cells of the glomerulas and that urinary levels of miR 192 and miR 200 are decreased in patients with bladder cancer, correlating with increased EMT (31).

In this study patients had normal renal function and therefore a change in renal function following surgery was not expected. However, other studies have reported improvements in renal function following bariatric procedures. Weight loss is associated with a reduction in proteinuria, including weight loss achieved surgically (7–9). Bariatric surgery improves renal function in patients with pre-existing glomerular hyperfiltration (9). Surgery also leads to reduced levels of albuminuria 1 year post-operatively, and levels continue to decrease up to 2 years and were maintained up to 5 years post-surgery (51, 52). Remission of microalbuminuria in individuals with diabetes can reach 60% 5 years following surgery. Surgery also has preventive effects, reducing the incidence of microalbuminuria by 80% and diabetic nephropathy by 60% compared to patients treated medically (52). Bariatric surgery can also lead to improvement and remission in patients with established CKD (53). The results presented here suggest miR 192 may mediate these renal improvements following bariatric surgery.

The early detection of DN is important in preventing progression to renal failure and there are considerable limitations of conventional markers of kidney function. Novel markers of DN have been reported, including proteins, peptides, and growth factors (54). Several studies have reported distinct microRNA profiles in urine, urinary sediments, and circulation in cases of kidney diseases. Urinary miR 29b, miR 29c, and miR 93 levels are correlated with proteinuria and renal scarring in patients with IgA nephropathy (55). Urinary microRNA profiles can also be used to differentiate various stages of diabetic nephropathy in individuals with type 1 diabetes (35). MicroRNA 145 is upregulated in the urinary exosomes of type 1 diabetes patients with microalbuminuria (56) and urinary microRNA levels, including levels of miR 192, can predict the development of microalbuminuria (57). In circulation, increased levels of miR 25, miR 27, miR 152, and miR 182 has been reported in patients with type 1 diabetes (58), and a recent report identified 10 elevated and two lowered microRNAs in patients with diabetes with DN relative to healthy controls (59). Circulating microRNAs can select diabetes patients with DN from those with good renal function, suggesting prognostic potential (59). This has led to interest in microRNAs as potential novel drug targets. The successful manipulation of miR 192 expression in the renal cortex of normal and diabetic mice (60) suggests the same may be possible in humans.

Other microRNAs have been reported to be involved in DN, including miR 21, miR 25, and miR 29 (61–63). The expression of more microRNAs needs to be assessed in bariatric urine samples and validated *in vitro*. However, there are limitations to using *in vitro* models of renal function, as EMT is more commonly observed in immortalized cell lines than *in vivo* renal fibrosis (64). The role of microRNAs in mediating the post-bariatric improvements in renal health needs to be confirmed in larger, prospective, multi-centre studies of bariatric patients including cohorts with varying degrees of kidney dysfunction.

In summary, bariatric surgery increased the urinary expression of the anti-fibrotic microRNAs miR 192 and miR 200 to levels found in the healthy controls. Several recent studies have assessed the suitability of urinary microRNAs as biomarkers, given the clinical ease of collecting and analyzing samples and the limitation of conventional markers of renal function. This study highlights the potential of urinary microRNAs as non-invasive biomarkers of health following bariatric surgery.

## Ethics Statement

This study was carried out in accordance with the recommendations of East Yorkshire and North Lincolnshire Research Ethics Committee and the Charing Cross Research Ethics Committee under ethics approval 09/H1304/46 and 08/H0711/123, with written informed consent from all subjects. All subjects gave written informed consent in accordance with the Declaration of Helsinki. The protocol was approved by the East Yorkshire and North Lincolnshire Research Ethics Committee and the Charing Cross Research Ethics Committee.

## Author Contributions

AA, HA, TS, SA, and NG contributed to the conceptual design of this study. AA conducted the experiments and data analysis. AA, HA, and NG interpreted the data and wrote the article. AA, HA, TS, AD, EH, TA, SA, and NG critical evaluated and approved the final article.

### Conflict of Interest Statement

The authors declare that the research was conducted in the absence of any commercial or financial relationships that could be construed as a potential conflict of interest.

## Supplementary Material

Supplementary Figure 1 Pearson correlation between the delta Ct of miR 192 and the delta Ct of **(A)** miR 200a and **(B)** miR 200b. *n* = 27 for miR 192 v miR 200a. *n* = 24 for miR 192 v miR 200b.

## References

[1] Maric-Bilkan C. Obesity and diabetic kidney disease. Med Clin North Am. (2013) 97:59–74. 10.1016/j.mcna.2012.10.010 23290730 PMC3539140

[2] Molitch ME DeFronzo RA Franz MJ Keane WF Mogensen CE Parving HH. Nephropathy in diabetes. Diabetes Care. (2004) 27(Suppl 1):S79–83. 10.2337/diacare.27.2007.S79 14693934

[3] Bowen T Jenkins RH Fraser DJ. MicroRNAs, transforming growth factor beta-1, and tissue fibrosis. J Pathol. (2013) 229:274–85. 10.1002/path.4119 23042530

[4] Gregory PA Bert AG Paterson EL Barry SC Tsykin A Farshid G. The miR-200 family and miR-205 regulate epithelial to mesenchymal transition by targeting ZEB1 and SIP1. Nat Cell Biol. (2008) 10:593–601. 10.1038/ncb1722 18376396

[5] Korpal M Lee ES Hu G Kang Y. The miR-200 family inhibits epithelial-mesenchymal transition and cancer cell migration by direct targeting of E-cadherin transcriptional repressors ZEB1 and ZEB2. J Biol Chem. (2008) 283:14910–4. 10.1074/jbc.C800074200 18411277 PMC3258899

[6] Krupa A Jenkins R Luo DD Lewis A Phillips A Fraser D. Loss of MicroRNA-192 promotes fibrogenesis in diabetic nephropathy. J Am Soc Nephrol. (2010) 21:438–47. 10.1681/ASN.2009050530 20056746 PMC2831862

[7] Chagnac A Weinstein T Herman M Hirsh J Gafter U Ori Y. The effects of weight loss on renal function in patients with severe obesity. J Am Soc Nephrol. (2003) 14:1480–6. 10.1097/01.ASN.0000068462.38661.89 12761248

[8] Morales E Valero MA Leon M Hernandez E Praga M. Beneficial effects of weight loss in overweight patients with chronic proteinuric nephropathies. Am J Kidney Dis. (2003) 41:319–27. 10.1053/ajkd.2003.50039 12552492

[9] Agnani S Vachharajani VT Gupta R Atray NK Vachharajani TJ. Does treating obesity stabilize chronic kidney disease? BMC Nephrol. (2005) 6:7. 10.1186/1471-2369-6-7 15955257 PMC1181818

[10] Ashrafian H Bueter M Ahmed K Suliman A Bloom SR Darzi A. Metabolic surgery: an evolution through bariatric animal models. Obes Rev. (2010) 11:907–20. 10.1111/j.1467-789X.2009.00701.x 20051020

[11] Angrisani L Santonicola A Iovino P Vitiello A Zundel N Buchwald H Bariatric surgery and endoluminal procedures: IFSO worldwide survey 2014. Obes Surg. (2017) 27:2279–89. 10.1007/s11695-017-2666-x 28405878 PMC5562777

[12] Wittgrove AC Clark GW Tremblay LJ. Laparoscopic gastric bypass, Roux-en-Y: preliminary report of five cases. Obes Surg. (1994) 4:353–7. 10.1381/096089294765558331 10742801

[13] Marceau P Biron S Bourque RA Potvin M Hould FS Simard S. Biliopancreatic diversion with a new type of gastrectomy. Obes Surg. (1993) 3:29–35. 10.1381/096089293765559728 10757900

[14] Salem L Jensen CC Flum DR. Are bariatric surgical outcomes worth their cost? A systematic review. J Am Coll Surg. (2005) 200:270–8. 10.1016/j.jamcollsurg.2004.09.045 15664103

[15] Sjostrom L Narbro K Sjostrom CD Karason K Larsson B Wedel H. Effects of bariatric surgery on mortality in Swedish obese subjects. N Engl J Med. (2007) 357:741–52. 10.1056/NEJMoa066254 17715408

[16] Pories WJ Swanson MS MacDonald KG Long SB Morris PG Brown BM. Who would have thought it? An operation proves to be the most effective therapy for adult-onset diabetes mellitus. Ann Surg. (1995) 222:339–50, discussion 350–332. 7677463 10.1097/00000658-199509000-00011 PMC1234815

[17] Buchwald H Estok R Fahrbach K Banel D Jensen MD Pories WJ. Weight and type 2 diabetes after bariatric surgery: systematic review and meta-analysis. Am J Med. (2009) 122:248–56 e245. 10.1016/j.amjmed.2008.09.041 19272486

[18] Mingrone G Panunzi S De Gaetano A Guidone C Iaconelli A Leccesi L. Bariatric surgery versus conventional medical therapy for type 2 diabetes. N Engl J Med. (2012) 366:1577–85. 10.1056/NEJMoa1200111 22449317

[19] Schauer PR Kashyap SR Wolski K Brethauer SA Kirwan JP Pothier CE. Bariatric surgery versus intensive medical therapy in obese patients with diabetes. N Engl J Med. (2012) 366:1567–76. 10.1056/NEJMoa1200225 22449319 PMC3372918

[20] Laferrere B Teixeira J McGinty J Tran H Egger JR Colarusso A. Effect of weight loss by gastric bypass surgery versus hypocaloric diet on glucose and incretin levels in patients with type 2 diabetes. J Clin Endocrinol Metab. (2008) 93:2479–85. 10.1210/jc.2007-2851 18430778 PMC2453054

[21] Fenske WK Pournaras DJ Aasheim ET Miras AD Scopinaro N Scholtz S. Can a protocol for glycaemic control improve type 2 diabetes outcomes after gastric bypass? Obes Surg. (2012) 22:90–6. 10.1007/s11695-011-0543-6 22052198

[22] Ashrafian H Athanasiou T Li JV Bueter M Ahmed K Nagpal K. Diabetes resolution and hyperinsulinaemia after metabolic Roux-en-Y gastric bypass. Obes Rev. (2011) 12:e257–272. 10.1111/j.1467-789X.2010.00802.x 20880129

[23] Ashrafian H Ahmed K Rowland SP Patel VM Gooderham NJ Holmes E. Metabolic surgery and cancer: protective effects of bariatric procedures. Cancer. (2010). 10.1002/cncr.25738 21509756

[24] Ashrafian H le Roux CW Rowland SP Ali M Cummin AR Darzi A. Metabolic surgery and obstructive sleep apnoea: the protective effects of bariatric procedures. Thorax. (2011) 67:442–9. 10.1136/thx.2010.151225 21709167

[25] Mitchell PS Parkin RK Kroh EM Fritz BR Wyman SK Pogosova-Agadjanyan EL. Circulating microRNAs as stable blood-based markers for cancer detection. Proc Natl Acad Sci USA. (2008) 105:10513–8. 10.1073/pnas.0804549105 18663219 PMC2492472

[26] Sharkey JW Antoine DJ Park BK. Validation of the isolation and quantification of kidney enriched miRNAs for use as biomarkers. Biomarkers. (2012) 17:231–9. 10.3109/1354750X.2012.657246 22356305

[27] Guay C Roggli E Nesca V Jacovetti C Regazzi R. Diabetes mellitus, a microRNA-related disease? Transl Res. (2011) 157:253–64. 10.1016/j.trsl.2011.01.009 21420036

[28] Ortega FJ Mercader JM Catalan V Moreno-Navarrete JM Pueyo N Sabater M. Targeting the circulating microRNA signature of obesity. Clin Chem. (2013) 59:781–92. 10.1373/clinchem.2012.195776 23396142

[29] Alkandari A Ashrafian H Sathyapalan T Sedman P Darzi A Holmes E. Improved physiology and metabolic flux after Roux-en-Y gastric bypass is associated with temporal changes in the circulating microRNAome: a longitudinal study in humans. BMC Obes. (2018) 5:20. 10.1186/s40608-018-0199-z 29881628 PMC5984421

[30] Wang G Kwan BC Lai FM Chow KM Kam-Tao Li P Szeto CC. Expression of microRNAs in the urinary sediment of patients with IgA nephropathy. Dis Markers. (2010) 28:79–86. 10.3233/DMA-2010-0687 20364043 PMC3833417

[31] Wang G Chan ES Kwan BC Li PK Yip SK Szeto CC. Expression of microRNAs in the urine of patients with bladder cancer. Clin Genitourin Cancer. (2012) 10:106–13. 10.1016/j.clgc.2012.01.001 22386240

[32] NICE Obesity: NICE Clinical Guideline 43. London: National Institute for Health and Care Excellence (NICE) (2006). Available online at: http://www.nice.org.uk/CG43

[33] Livak KJ Schmittgen TD. Analysis of relative gene expression data using real-time quantitative PCR and the 2(-Delta Delta C(T)) Method. Methods. (2001) 25:402–8. 10.1006/meth.2001.1262 11846609

[34] Sun Y Koo S White N Peralta E Esau C Dean NM. Development of a micro-array to detect human and mouse microRNAs and characterization of expression in human organs. Nucleic Acids Res. (2004) 32:e188. 10.1093/nar/gnh186 15616155 PMC545483

[35] Argyropoulos C Wang K McClarty S Huang D Bernardo J Ellis D. Urinary microRNA profiling in the nephropathy of type 1 diabetes. PLoS ONE. (2013) 8:e54662. 10.1371/journal.pone.0054662 23358711 PMC3554645

[36] Sathyapalan T David R Gooderham NJ Atkin SL. Increased expression of circulating miRNA-93 in women with polycystic ovary syndrome may represent a novel, non-invasive biomarker for diagnosis. Sci Rep. (2015) 5:16890. 10.1038/srep16890 26582398 PMC4652283

[37] Harling L Lambert J Ashrafian H Darzi A Gooderham NJ Athanasiou T. Elevated serum microRNA 483-5p levels may predict patients at risk of post-operative atrial fibrillation. Eur J Cardiothorac Surg. (2017) 51:73–8. 10.1093/ejcts/ezw245 27422887 PMC5226070

[38] Simpson K Wonnacott A Fraser DJ Bowen T. MicroRNAs in diabetic nephropathy: from biomarkers to therapy. Curr Diab Rep. (2016) 16:35. 10.1007/s11892-016-0724-8 26973290 PMC4791477

[39] Harvey SJ Jarad G Cunningham J Goldberg S Schermer B Harfe BD. Podocyte-specific deletion of dicer alters cytoskeletal dynamics and causes glomerular disease. J Am Soc Nephrol. (2008) 19:2150–8. 10.1681/ASN.2008020233 18776121 PMC2573015

[40] Ho J Ng KH Rosen S Dostal A Gregory RI Kreidberg JA. Podocyte-specific loss of functional microRNAs leads to rapid glomerular and tubular injury. J Am Soc Nephrol. (2008) 19:2069–75. 10.1681/ASN.2008020162 18832437 PMC2573018

[41] Shi S Yu L Chiu C Sun Y Chen J Khitrov G. Podocyte-selective deletion of dicer induces proteinuria and glomerulosclerosis. J Am Soc Nephrol. (2008) 19:2159–69. 10.1681/ASN.2008030312 18776119 PMC2573016

[42] Wang B Herman-Edelstein M Koh P Burns W Jandeleit-Dahm K Watson A. E-cadherin expression is regulated by miR-192/215 by a mechanism that is independent of the profibrotic effects of transforming growth factor-beta. Diabetes. (2010) 59:1794–802. 10.2337/db09-1736 20393144 PMC2889781

[43] Wang B Koh P Winbanks C Coughlan MT McClelland A Watson A. miR-200a Prevents renal fibrogenesis through repression of TGF-beta2 expression. Diabetes. (2011) 60:280–7. 10.2337/db10-0892 20952520 PMC3012183

[44] Oba S Kumano S Suzuki E Nishimatsu H Takahashi M Takamori H. miR-200b precursor can ameliorate renal tubulointerstitial fibrosis. PLoS ONE. (2010) 5:e13614. 10.1371/journal.pone.0013614 21049046 PMC2963611

[45] Kim T Veronese A Pichiorri F Lee TJ Jeon YJ Volinia S. p53 regulates epithelial-mesenchymal transition through microRNAs targeting ZEB1 and ZEB2. J Exp Med. (2011) 208:875–83. 10.1084/jem.20110235 21518799 PMC3092351

[46] Park SM Gaur AB Lengyel E Peter ME. The miR-200 family determines the epithelial phenotype of cancer cells by targeting the E-cadherin repressors ZEB1 and ZEB2. Genes Dev. (2008) 22:894–907. 10.1101/gad.1640608 18381893 PMC2279201

[47] Kato M Arce L Wang M Putta S Lanting L Natarajan R. A microRNA circuit mediates transforming growth factor-beta1 autoregulation in renal glomerular mesangial cells. Kidney Int. (2011) 80:358–68. 10.1038/ki.2011.43 21389977 PMC3337779

[48] Kato M Zhang J Wang M Lanting L Yuan H Rossi JJ. MicroRNA-192 in diabetic kidney glomeruli and its function in TGF-beta-induced collagen expression via inhibition of E-box repressors. Proc Natl Acad Sci USA. (2007) 104:3432–7. 10.1073/pnas.0611192104 17360662 PMC1805579

[49] Putta S Lanting L Sun G Lawson G Kato M Natarajan R. Inhibiting microRNA-192 ameliorates renal fibrosis in diabetic nephropathy. J Am Soc Nephrol. (2012) 23:458–69. 10.1681/ASN.2011050485 22223877 PMC3294315

[50] Tian Z Greene AS Pietrusz JL Matus IR Liang M. MicroRNA-target pairs in the rat kidney identified by microRNA microarray, proteomic, and bioinformatic analysis. Genome Res. (2008) 18:404–11. 10.1101/gr.6587008 18230805 PMC2259104

[51] Navarro-Diaz M Serra A Romero R Bonet J Bayes B Homs M. Effect of drastic weight loss after bariatric surgery on renal parameters in extremely obese patients: long-term follow-up. J Am Soc Nephrol. (2006) 17(12 Suppl 3):S213–7. 10.1681/ASN.2006080917 17130264

[52] Heneghan HM Cetin D Navaneethan SD Orzech N Brethauer SA Schauer PR. Effects of bariatric surgery on diabetic nephropathy after 5 years of follow-up. Surg Obes Relat Dis. (2013) 9:7–14. 10.1016/j.soard.2012.08.016 23211651

[53] Alexander JW Goodman HR Hawver LR Cardi MA. Improvement and stabilization of chronic kidney disease after gastric bypass. Surg Obes Relat Dis. (2009) 5:237–41. 10.1016/j.soard.2008.08.016 18996757

[54] Fassett RG Venuthurupalli SK Gobe GC Coombes JS Cooper MA Hoy WE. Biomarkers in chronic kidney disease: a review. Kidney Int. (2011) 80:806–21. 10.1038/ki.2011.198 21697815

[55] Wang G Kwan BC Lai FM Chow KM Li PK Szeto CC. Urinary miR-21, miR-29, and miR-93: novel biomarkers of fibrosis. Am J Nephrol. (2012) 36:412–8. 10.1159/000343452 23108026

[56] Barutta F Tricarico M Corbelli A Annaratone L Pinach S Grimaldi S. Urinary exosomal microRNAs in incipient diabetic nephropathy. PLoS ONE. (2013) 8:e73798. 10.1371/journal.pone.0073798 24223694 PMC3817183

[57] Argyropoulos C Wang K Bernardo J Ellis D Orchard T Galas D. Urinary microRNA profiling predicts the development of microalbuminuria in patients with type 1 diabetes. J Clin Med. (2015) 4:1498–517. 10.3390/jcm4071498 26239688 PMC4519802

[58] Nielsen LB Wang C Sorensen K Bang-Berthelsen CH Hansen L Andersen ML. Circulating levels of microRNA from children with newly diagnosed type 1 diabetes and healthy controls: evidence that miR-25 associates to residual beta-cell function and glycaemic control during disease progression. Exp Diabetes Res. (2012) 2012:896362. 10.1155/2012/896362 22829805 PMC3398606

[59] Bijkerk R Duijs JM Khairoun M Ter Horst CJ van der Pol P Mallat MJ. Circulating microRNAs associate with diabetic nephropathy and systemic microvascular damage and normalize after simultaneous pancreas-kidney transplantation. Am J Transplant. (2015) 15:1081–90. 10.1111/ajt.13072 25716422

[60] Kato M Natarajan R. Diabetic nephropathy–emerging epigenetic mechanisms. Nat Rev Nephrol. (2014) 10:517–30. 10.1038/nrneph.2014.116 25003613 PMC6089507

[61] Fu Y Zhang Y Wang Z Wang L Wei X Zhang B. Regulation of NADPH oxidase activity is associated with miRNA-25-mediated NOX4 expression in experimental diabetic nephropathy. Am J Nephrol. (2010) 32:581–9. 10.1159/000322105 21071935

[62] Dey N Das F Mariappan MM Mandal CC Ghosh-Choudhury N Kasinath BS. MicroRNA-21 orchestrates high glucose-induced signals to TOR complex 1, resulting in renal cell pathology in diabetes. J Biol Chem. (2011) 286:25586–603. 10.1074/jbc.M110.208066 21613227 PMC3138272

[63] Wang B Komers R Carew R Winbanks CE Xu B Herman-Edelstein M. Suppression of microRNA-29 expression by TGF-beta1 promotes collagen expression and renal fibrosis. J Am Soc Nephrol. (2012) 23:252–65. 10.1681/ASN.2011010055 22095944 PMC3269175

[64] Kriz W Kaissling B Le Hir M Epithelial-mesenchymal transition (EMT) in kidney fibrosis: fact or fantasy? J Clin Invest. (2011) 121:468–74. 10.1172/JCI44595 21370523 PMC3026733

[65] Alkandari A Ashrafian H Sathyapalan T Efthimiou E Darzi A Holmes E Urinary microRNAs as non-invasive biomarkers of surgery-induced improvement of renal function. In: Abstracts of the 21st Pan Arab Conference on Diabetes PACD21, March 2017 and the 6th Middle East Congress on Clinical Nutrition, March 2017. Cairo: Primary Care Diabetes (2017).

