# Supplementary figures and images for: Bariatric Surgery Modulates Urinary Levels of MicroRNAs Involved in the Regulation of Renal Function
Source: Front Endocrinol (Lausanne). 2019 May 21;10:319. doi: 10.3389/fendo.2019.00319 (PMC6536704; doi:10.3389/fendo.2019.00319)

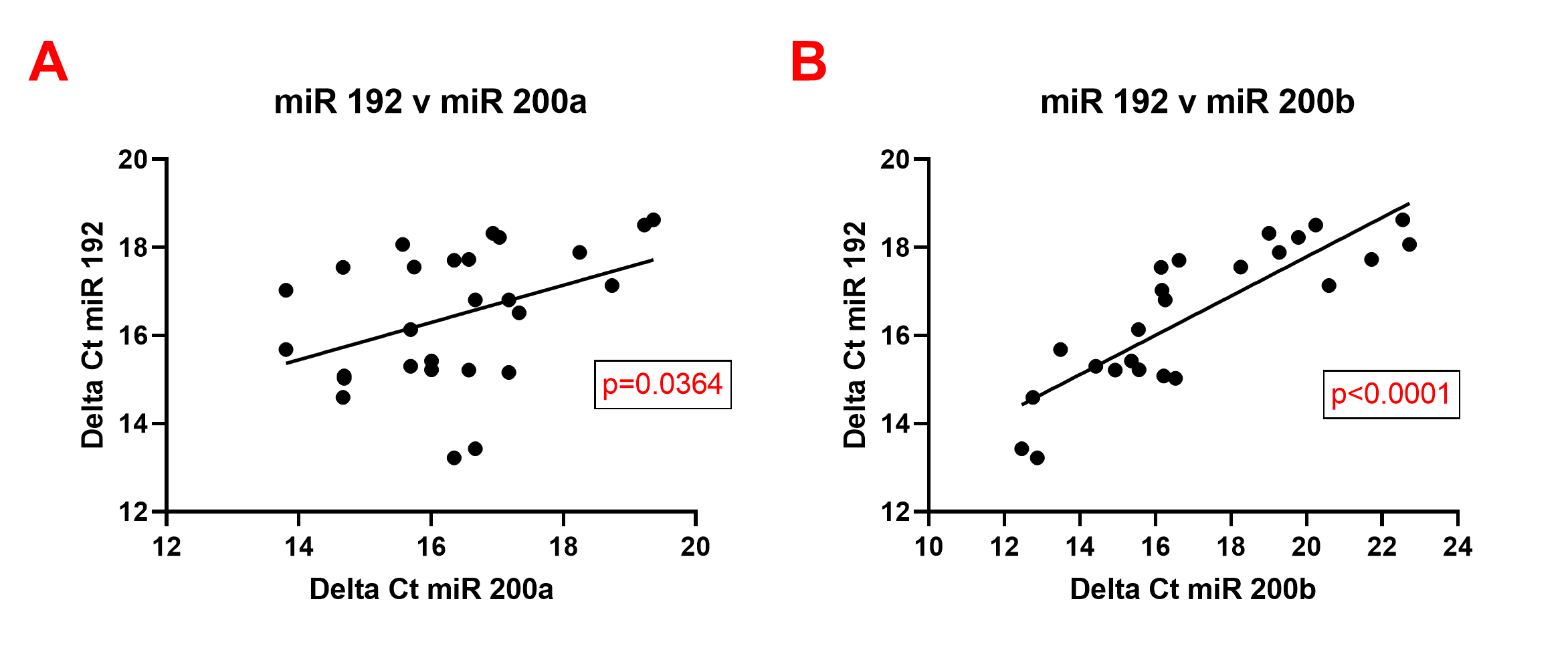

Supplement: Supplementary Figure 1 — Pearson correlation between the delta Ct of miR 192 and the delta Ct of (A) miR 200a and (B) miR 200b. n = 27 for miR 192 v miR 200a. n = 24 for miR 192 v miR 200b. [file Image_1.TIF]
